# Barriers and Needs for Postpartum Contraception

**DOI:** 10.1007/s10995-026-04265-0

**Published:** 2026-05-04

**Authors:** Amanda Spishak-Thomas, Erica L. Eliason

**Affiliations:** 1 Center for State Health Policy, Rutgers University, New Brunswick, USA; 2 Department of Urban-Global Public Health, Rutgers School of Public Health, Piscataway, USA

**Keywords:** Contraception, Maternal health, Postpartum, Family planning

## Abstract

**Objectives:**

Family planning after childbirth can address postpartum individuals’ contraceptive needs and preferences for birth spacing. Our study explored the reasons for not using postpartum contraception and identified opportunities to remove contraceptive barriers.

**Methods:**

This study used the 2019–2021 Pregnancy Risk Assessment Monitoring System survey of individuals with a recent live birth in 40 states, New York City, and the District of Columbia. We used mixed-methods to analyze categorical and free-text responses on the reasons for not using postpartum contraception. Descriptive and bivariate analysis were applied to characterize the data and explain the relationship between variables. Qualitative data was processed by manual coding procedures.

**Results:**

Among the 115,917 sample respondents, 22.9% reported not using contraception in the postpartum period. The most common responses were not wanting to use birth control (39.3%) and concerns about contraceptive side effects (32.2%). There were 4,508 postpartum people who completed a free-text response and primarily reported not using contraception due to difficulties with appointment processes and/or not enough time, and infertility or trouble conceiving.

**Conclusions for Practice:**

We find that postpartum people predominantly reported not wanting to use birth control, contraceptive side effects, infertility, and appointment processes, as reasons for not taking actions to prevent pregnancy. These findings suggest a need for additional counseling about side effects and benefits of contraception and can inform policies and practices that promote reproductive health equity in the postpartum period.

**Supplementary Information:**

The online version contains supplementary material available at 10.1007/s10995-026-04265-0.

## Significance

Birthing people are more engaged in the health care system during pregnancy but still face health systems barriers that prevent family planning access in the postpartum period. Postpartum family planning services are important for reducing rapid repeat pregnancies, which are associated with adverse maternal and perinatal outcomes. Dismantling health systems barriers by improving postpartum contraception access also promotes reproductive autonomy. We identify the prevalence of barriers to postpartum contraceptive use and associated demographic characteristics. Our findings can inform provider practices and policies regarding access to reproductive health care, contraception, and family planning services.

**Supplementary Information:**

The online version contains supplementary material available at 10.1007/s10995-026-04265-0.

## Introduction

The postpartum period presents a unique opportunity to address the needs of birthing people, who are more likely to be engaged in the healthcare system during pregnancy but have high rates of insurance transitions after childbirth compared to pregnancy (Daw et al., 2017). Despite elevated perinatal healthcare engagement, many birthing people face health systems barriers that prevent family planning access in the postpartum period (Holden et al., 2018; Zerden et al., 2015). Increasing access to postpartum family planning services is important for promoting birth spacing and reducing rapid repeat pregnancies, which occur within short intervals after childbirth (Appareddy et al., 2017; Dee, 2017; Hutcheon et al., 2019; Schummers et al., 2018). Short interpregnancy intervals are associated with increased risks of adverse maternal and perinatal outcomes such as precipitous labor, uterine rupture, maternal mortality, severe morbidity, and spontaneous preterm delivery (Appareddy et al., 2017; Hutcheon et al., 2019; Schummers et al., 2018). Improving pregnancy spacing is a Healthy People 2030 goal in the U.S., with recommendations for improving family planning services and counseling on contraceptives after childbirth to achieve these goals (Department of Health and Human Services, n.d.).

The 2010 Affordable Care Act (ACA) drastically changed the healthcare environment in the U.S., with millions of reproductive age people gaining insurance (Johnston et al., 2018). Several components of the ACA have the potential to improve postpartum access to family planning services, including insurance through expanded Medicaid, Marketplace, or the dependent coverage provision (Darney et al., 2020; Eliason, 2019; Eliason et al., 2022; Gordon et al., 2020; Moniz et al., 2018; Patton et al., 2019). With the various ACA provisions in play, it is important to examine current barriers to contraceptive access that postpartum people face and identify opportunities to improve access to contraceptive services.

Improved postpartum contraceptive access is essential for advancing reproductive justice, which involves dismantling systemic barriers to care (Chiang et al., 2026; Gilliam et al., 2009; McAllister et al., 2023). To promote reproductive health equity, it is necessary to ensure that individuals have the opportunity to choose from the full spectrum of contraceptive methods (McAllister et al., 2023). The COVID-19 pandemic exacerbated disparities and limited access to healthcare, including perinatal care and contraception, making the need for targeted interventions more urgent (Brislane et al., 2021; Czeisler et al., 2020; Eliason et al., 2023; Steenland et al., 2022). Identifying the reasons why postpartum people are not using family planning methods can reveal unmet needs for contraception, barriers to services, and areas for intervention to improve contraceptive access and promote reproductive justice. This study aims to both quantitatively and qualitatively identify the reasons for not using postpartum contraception along with associated demographic characteristics.

## Materials and Methods

### Data and Sample

This study used 2019–2021 Pregnancy Risk Assessment Monitoring System (PRAMS) data, a multi-state survey of individuals with a recent live birth conducted by the Centers for Disease Control and Prevention (Shulman et al., 2018). Participating states draw samples from birth certificate files, with questionnaires completed 2–6 months after birth. This study included 40 participating states, New York City, and the District of Columbia that met survey response rate minimum thresholds for PRAMS (Supplementary Material 1). We weighted all data using PRAMS sample weights, which accounts for sample stratification and survey non-response. This study was determined to be non-human subject research by the Rutgers University institutional review board.

### Variables

Our outcomes of interest were reasons underlying contraceptive decisions in the postpartum period. Reasons were identified through responses to two questions, first, whether respondents were doing anything to keep from getting pregnant at the time of the survey, and second, what the reasons were for not doing anything. Respondents were asked to select all that applied, with a free-text option if reasons fell outside given categories. Categorical response options for not taking actions to prevent pregnancy included: (1) abstinence, (2) wanting to get pregnant, (3) not wanting to use birth control, (4) a husband or partner not wanting to use anything, (5) problems paying for birth control, (6) concern about side effects from birth control, (7) currently pregnant, (8) blocked or tied tubes, and (9) other. Consistent with prior work that found PRAMS respondents frequently reported reasons for nonuse outside of the provided categorical options (Richards et al., 2022), we qualitatively analyzed free-text responses using content analysis and manual coding procedures.

We examined demographic characteristics, which included age (19 or younger, 20–24, 25–29, 30–34, 35–39, 40+), educational attainment (high school education or less, more than high school education), race and ethnicity (non-Hispanic white, non-Hispanic Black, Hispanic/Latino, non-Hispanic Asian/Pacific Islander, non-Hispanic American Indian or Alaska Native, other), marital status (married, not married), number of prior births (0, 1, 2, 3 or more), postpartum health insurance (private, Medicaid/CHIP, uninsured, other), and household poverty status (138% FPL or below, 139%–200% FPL, above 200% FPL). We calculated federal poverty level (FPL) based on household size and income, using the midpoint of the categorical income variable. We created missing categories for all demographic variables with missing data (White & Thompson, 2005).

### Statistical Analysis

We calculated the proportion of respondents reporting each reason for not using postpartum contraception under the categorical response options. For the qualitative data analysis, we used thematic analysis techniques (Braun & Clarke, 2006), in which we reviewed and coded free-text responses to form categories based on reasons for not using postpartum contraception. Concurrently, we employed inductive and deductive approaches to enhance the robustness of findings (Miles et al., 2018). We identified the key themes (i.e., inductive analytical approach) and mapped them to our original hypotheses (i.e., deductive approach) guided by the literature and existing PRAMS categories. We omitted free-text responses in a language other than English (*n* = 188).

To identify the individual factors associated with not using postpartum contraception, we generated odds ratios using survey-weighted multivariable logistic regression models. We included marginal effects of logistic regression models as predicted probabilities in the appendix (Supplementary Material 2). We conducted a sensitivity analysis using insurance at delivery instead of postpartum insurance to ensure our results were not sensitive to insurance transitions (Supplementary Material 3). We additionally examined the proportions of the study sample not using postpartum contraception across demographic characteristics (Supplementary Material 4). Further, we conducted a sensitivity analysis of main models including state fixed effects and state-clustered standard errors to account for state-level variation (Supplementary Material 5). Finally, we stratified analyses by postpartum health insurance to understand if demographic characteristics associated with not using postpartum contraception differ from the overall sample (Supplementary Material 6). All analyses were conducted in 2025 and 2026 using Stata v.18.5.

## Results

The sample included 115,917 respondents, representing a weighted total of 6,100,594 postpartum people residing in the 42 study jurisdictions. Table 1 shows the survey-weighted demographic characteristics of the sample by postpartum contraceptive use. Among the 115,917 postpartum people, 26,492 respondents (22.9%) reported not taking any actions to prevent pregnancy. There were statistically significant differences in the proportions of the demographic characteristics by postpartum contraceptive use. Postpartum people not using contraception had slightly lower rates of marriage (59.3% vs. 61.8%), were more likely to be non-Hispanic (NH) Black (17.8% vs. 14.3%) and had slightly lower rates of educational attainment beyond high school (61.0% vs. 64.3%). The age distribution of those using and not using contraception were similar with most either between the age of 30 and 34 (28.5% vs. 31.2%) or 25 and 29 (27.1% vs. 28.7%). Similarly, income levels were comparable, with incomes at or below 138% of the FPL among 29.6% of respondents not using contraception (vs. 29.3% among respondents using contraception). Postpartum people not using contraception had slightly lower rates of private insurance (49.0% vs. 52.1%) and slightly higher rates of no prior birth (43.7% vs. 38.3%).

**Table 1:** Characteristics of the study sample of postpartum people, by postpartum contraceptive use, 2019–2021

Demographic characteristics	Postpartum people not using contraception (*n* = 26,492)		Postpartum people using contraception (*n* = 89,425)		*P* value of difference
	Unweighted sample size	Weighted proportion	Unweighted sample size	Weighted proportion	
*Age (in years)*					< 0.001
19 or younger	1,065	3.81 (3.45–4.20)	3,601	3.97 (3.76–4.19)	
20–24	4,626	18.22 (17.44–19.02)	14,914	17.12 (16.72–17.54)	
25–29	7,060	27.07 (26.22–27.94)	25,228	28.69 (28.21–29.18)	
30–34	7,715	28.47 (27.61–29.34)	28,070	31.23 (30.75–31.72)	
35–39	4,713	17.77 (17.05–18.50)	14,597	15.85 (15.48–16.24)	
40+	1,312	4.67 (4.29–5.08)	3,009	3.13 (2.95–3.31)	
*Educational attainment*					< 0.001
High school education or less	9,852	38.2 (37.25–39.16)	30,460	34.98 (34.47–35.50)	
More than high school education	16,401	60.97 (60.00–61.92)	58,356	64.28 (63.77–64.79)	
*Race/ethnicity*					< 0.001
White, non-Hispanic	10,757	52.35 (51.40–53.29)	42,737	58.17 (57.68–58.66)	
Black, non-Hispanic	5,318	17.82 (17.12–18.55)	14,674	14.26 (13.94–14.60)	
Hispanic or Latino	4,094	16.43 (15.71–17.18)	15,096	17.89 (17.49–18.30)	
Asian or Pacific Islander	2,816	7.29 (6.87–7.75)	5,856	4.49 (4.31–4.68)	
American Indian or Alaska Native	1,149	0.78 (0.68–0.89)	3,070	0.64 (0.59–0.69)	
Other	1,667	3.77 (3.42–4.15)	5,218	3.05 (2.88–3.22)	
*Marital status*					< 0.001
Married	15,202	59.25 (58.28–60.20)	54,449	61.84 (61.32–62.35)	
Not Married	11,271	40.66 (39.71–41.63)	34,918	38.12 (37.61–38.63)	
*Number of prior births*					< 0.001
0	11,515	43.72 (42.76–44.69)	34,256	38.28 (37.77–38.80)	
1	7,840	30.39 (29.51–31.28)	28,271	32.84 (32.34–33.34)	
2	3,780	14.16 (13.50–14.85)	15,223	17.01 (16.61–17.41)	
3 or more	3,310	11.54 (10.94–12.17)	11,531	11.69 (11.35–12.03)	
*Postpartum health insurance*					< 0.001
Private	12,294	49.04 (48.07–50.00)	44,804	52.05 (51.53–52.57)	
Medicaid or CHIP	10,092	35.92 (35.00–36.85)	31,819	33.73 (33.24–34.22)	
Uninsured	2,522	8.96 (8.39–9.56)	7,963	8.44 (8.14–8.76)	
Other	1,161	4.48 (4.10–4.89)	3,605	4.35 (4.13–4.57)	
*Income*					< 0.01
138% FPL and below	8,456	29.62 (28.74–30.52)	27,529	29.33 (28.85–29.82)	
139%−200% FPL	2,516	9.35 (8.81–9.93)	8,970	9.57 (9.26–9.88)	
Above 200% FPL	5,859	21.54 (20.76–22.34)	21,171	23.15 (22.71–23.60)	

*Notes*: Authors’ analysis of 2019–2021 Pregnancy Risk Assessment Monitoring System data from 40 states, New York City, and the District of Columbia. Unweighted sample sizes and weighted proportions are presented. 95% confidence intervals are in parentheses. Pearson’s chi-squared tests were used to assess whether the demographic characteristics differed between postpartum people not using contraception and postpartum people using contraception

**Fig. 1:**
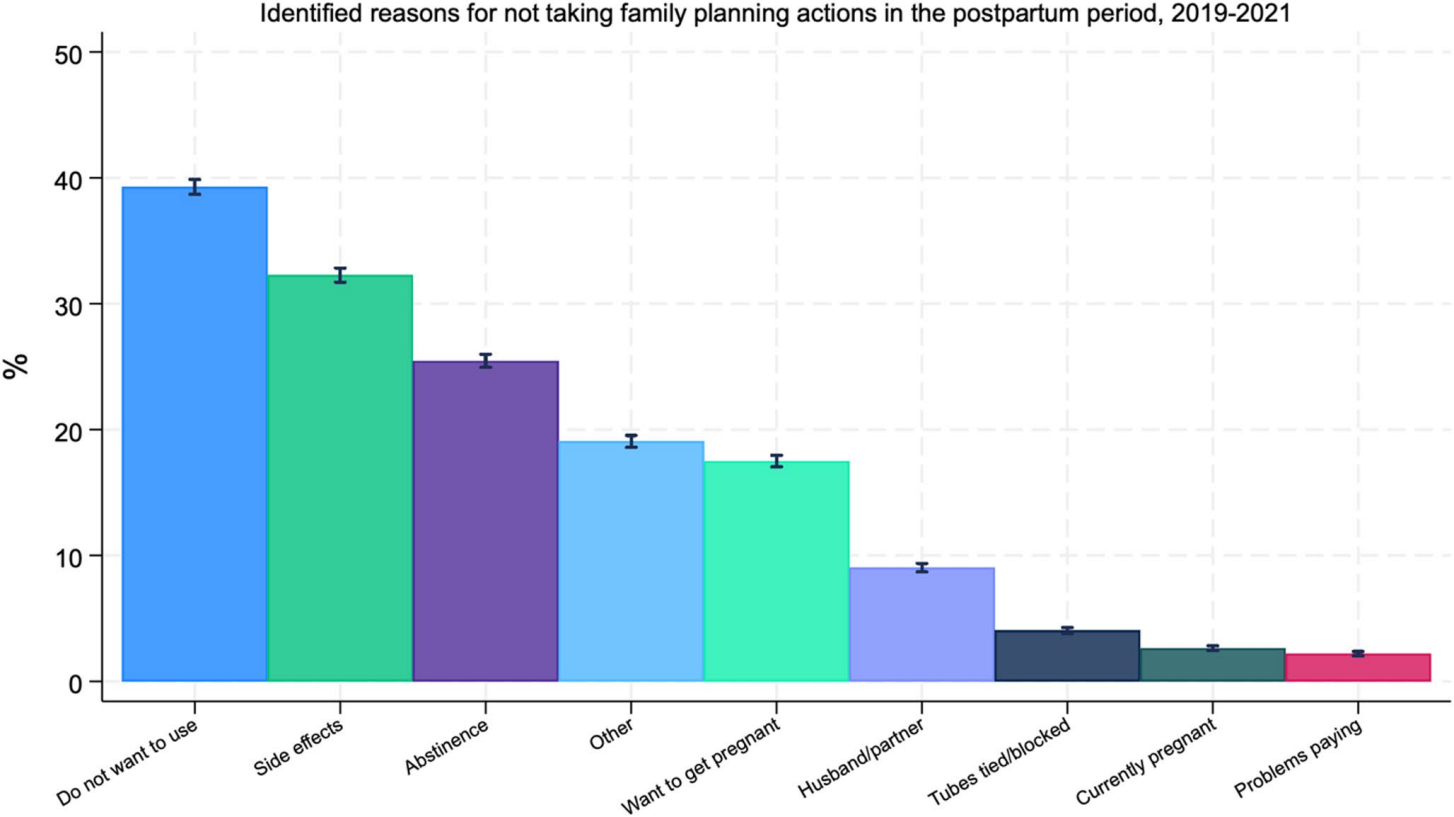
Identified reasons for not taking postpartum family planning actions. Authors’ analysis of 2019–2021 Pregnancy Risk Assessment Monitoring System data from 40 states, New York City, and the District of Columbia. *N* = 26,492. Weighted proportions are presented. Responses are not mutually exclusive. Error bars represent 95% CIs

Figure 1 displays postpartum respondents’ identified reasons for not taking family planning actions. Of the 26,492 people not using contraception, the most commonly reported reason (39.3%) was not wanting to use birth control, followed by concerns about side effects from birth control (32.2%). Abstinence was reported by a quarter of postpartum people (25.2%), followed by wanting to get pregnant (18%), and a small share reported currently being pregnant (2.8%). Problems paying for birth control were reported by under 2% of respondents, while a husband/partner not wanting to use contraception was reported by just under 10% of respondents.

**Fig. 2:**
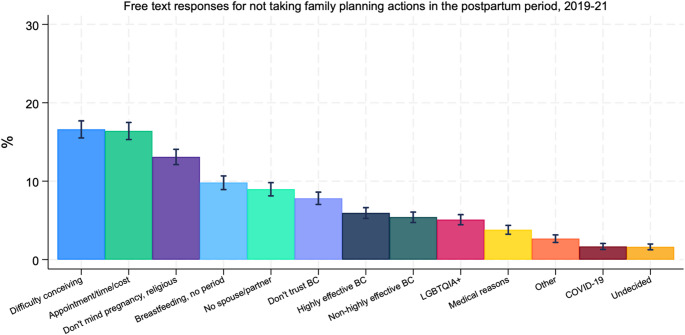
Free text responses for not taking postpartum family planning actions. Authors’ analysis of 2019–2021 Pregnancy Risk Assessment Monitoring System data from 40 states, New York City, and the District of Columbia. Free-text responses (*N* = 4,508) were provided for birthing people identifying “other” as the reason for not taking family planning actions in the postpartum period. Spanish responses were excluded from analysis (*N* = 180) as well as 8 numerical responses. Error bars represent 95% CIs

Reasons other than the categorical responses comprised about 20% of reported reasons for not using postpartum contraception between 2019 and 2021, with 4,508 free-text responses for qualitative analysis (Fig. 2). Among the free-text responses, the primary reason for not using contraception was related to difficulty conceiving, including infertility, age, reliance on in vitro fertilization, or polycystic ovary syndrome (16.6%), followed closely by the appointment process including time restrictions, cost, transportation, and health insurance (16.4%). Appointment-related reasons included that the “doctor didn’t send in prescription, and I don’t have anyone to watch my kids to go back to the doctor,” “waiting for IUD appointment,” “I just haven’t had time,” and “trouble getting to the doctor.”

The next most common responses were religious respondents who would not mind becoming pregnant again (13.1%). These responses included, “children are a blessing,” “whatever happens happens,” and “religious objections to any form of birth control.” Breastfeeding and/or not yet having a period comprised 9.8% of responses, and nearly 9% cited not having a spouse/partner. Among those who mentioned breastfeeding, many expressed concerns of birth control related to milk supply including, “the mini pill suppresses my milk production,” and “do not want birth control to decrease my milk supply” in addition to many reporting that birth control was unnecessary while breastfeeding “breastfeeding helps serve as birth control.”

Another frequent response was related to a history of side effects and dislike or distrust of birth control comprising 7.8% of free-text responses. Almost 6% of respondents reported using a highly effective birth control method ( e.g., prescriptions, hysterectomy, vasectomy, tubal ligation, or intrauterine device (IUD) and 5.4% reported using a non-highly effective method (e.g., abstinence, the pull-out method, natural family planning, or condoms). Around 5% of respondents reported being in same sex relationships where birth control was unnecessary and 3.8% described a medical reason limiting their ability to use contraception. A small share of respondents described the pandemic as being a barrier to family planning (1.7%), however this category only represents free-text responses from 2020 to 2021. Several COVID-19 responses described an inability to schedule surgical sterilization or a vasectomy due to the pandemic or lack of appointment availability and others felt scared going to a doctor, “afraid of going to doctor’s because of COVID.”

Table 2 presents multivariable logistic regression results of the characteristics associated with not using postpartum contraception by pandemic period. Across both periods, respondents with a high school education or less were significantly less likely to use postpartum contraception compared to respondents with higher educational attainment (2019 aOR: 1.3, 95% CI 1.1–1.5; 2020–2021 aOR: 1.2, 95% CI 1.1–1.3). During the pandemic, the adjusted odds of not using postpartum contraception were 1.4 (95% CI 1.3–1.5) times higher among NH-Black respondents, 1.4 (95% CI 1.1–1.7) times higher among American Indian or Alaska Native respondents, and 1.7 (95% CI 1.6–1.9) times higher among Asian or Pacific Islander respondents compared with NH-white respondents. In 2019, the adjusted odds of not using postpartum contraception were 1.3 (95% CI 1.1–1.5) times higher among NH-Black respondents, 1.7 (95% CI 1.5–2.1) times higher among Asian or Pacific Islander respondents, and 0.8 (95% CI 0.7–0.9) times lower among Hispanic or Latina respondents compared with NH-white respondents.

**Table 2:** Characteristics associated with not taking family planning actions in the postpartum period, 2019–2021

Demographic characteristics	2019 (*N* = 27,251)	2020–2021 (*N* = 88,664)
	Odds ratio	Odds ratio
*Age*		
19 or younger	Reference	Reference
20–24	1.17	1.41***
	(0.87–1.56)	(1.21–1.63)
25–29	1.17	1.43***
	(0.87–1.56)	(1.23–1.66)
30–34	1.17	1.46***
	(0.87–1.58)	(1.25–1.70)
35–39	1.65**	1.78***
	(1.21–2.25)	(1.52–2.08)
40+	2.05***	2.38***
	(1.41–2.98)	(1.97–2.87)
*Educational attainment*		
More than high school	Reference	Reference
High school education or less	1.28***	1.20***
	(1.13–1.45)	(1.12–1.28)
*Race/ethnicity*		
White, non-Hispanic	Reference	Reference
Black, non-Hispanic	1.29***	1.36***
	(1.11–1.49)	(1.26–1.46)
Hispanic or Latino	0.80**	1.00
	(0.69–0.94)	(0.92–1.08)
Asian or Pacific Islander	1.74***	1.72***
	(1.46–2.08)	(1.56–1.89)
American Indian or Alaska Native	1.02	1.37**
	(0.78–1.35)	(1.13–1.66)
Other	1.61***	1.29***
	(1.23–2.10)	(1.13–1.47)
*Marital status*		
Married	Reference	Reference
Not married	0.99	1.04
	(0.87–1.13)	(0.97–1.11)
Number of prior births		
0	Reference	Reference
1	0.75***	0.79***
	(0.66–0.84)	(0.74–0.84)
2	0.73***	0.65***
	(0.63–0.85)	(0.60–0.70)
3 or more	0.67***	0.74***
	(0.56–0.79)	(0.67–0.81)
*Postpartum health insurance*		
Private	Reference	Reference
Medicaid or CHIP	1.14	1.15***
	(0.98–1.33)	(1.07–1.24)
Uninsured	1.31**	1.15*
	(1.08–1.58)	(1.02–1.29)
Other	1.18	1.09
	(0.92–1.51)	(0.95–1.24)
Income		
138% FPL and below	Reference	Reference
139%−200% FPL	0.96	1.01
	(0.80–1.15)	(0.92–1.11)
Above 200% FPL	0.96	1.02
	(0.82–1.13)	(0.94–1.11)

*Notes*: Authors’ analysis of 2019–2021 Pregnancy Risk Assessment Monitoring System data from 40 states, New York City, and the District of Columbia. Weighted odds ratios presented with robust 95% confidence intervals

*p < 0.05, **p < 0.01, ***p < 0.001

By age, individuals 40 and older had the highest odds of not using postpartum contraception (2019 aOR: 2.1, 95% CI 1.4–3.0; 2020–2021 aOR: 2.4, 95% CI 2.0–2.9) compared with respondents 19 years and younger. Across the periods, respondents who had previous births had significantly lower odds of not using postpartum contraception compared to primiparous respondents. In 2020–2021, respondents with postpartum Medicaid or CHIP (aOR: 1.2, 95% CI 1.1–1.2) and uninsured respondents (aOR: 1.2, 95% CI 1.0–1.3) had higher odds of not using contraception compared with privately insured respondents. In 2019, only uninsured respondents had higher odds of not using postpartum contraception by insurance. The odds of not using postpartum contraception were not statistically significantly different by marital status or across federal poverty levels for either period.

Supplementary Material 2 presents the marginal effects for the logistic regression model. In analyses including insurance at delivery rather than postpartum insurance, we observed similar patterns in demographic characteristics associated with higher probabilities of nonuse of postpartum contraception as in main models (Supplementary Material 3). Examining rates of not using postpartum contraception across sample characteristics, we found higher rates among birthing people who had educational attainment of a high school education or less, were age 40 or older at delivery, were non-Hispanic Asian or Pacific Islander, were unmarried, had no previous births, and had postpartum health insurance that was not private coverage (Supplementary Material 4). In sensitivity analyses accounting for state-level variation, we found results that showed similar patterns of association between demographics and not taking family planning actions in the postpartum period as in main models (Supplementary Material 5). In stratified models by postpartum insurance, we observe results that showed similar patterns of association between demographic characteristics and not taking family planning actions as in main models, especially among Medicaid and privately insured respondents (Supplementary Material 6).

## Discussion

In this mixed methods analysis of PRAMS data from 42 jurisdictions, we found the main reasons postpartum people were not using contraception included not wanting to use birth control, concerns about contraceptive side effects, and reliance on abstinence. Among free-text responses, the main reasons were appointment processes and time, and infertility or difficulty with conception. During the pandemic, we identified 75 respondents who avoided doctor visits or were unable to schedule procedures such as a partner’s vasectomy or IUD due to COVID-19 related fears, which likely exacerbated appointment barriers. Demographic characteristics associated with not using postpartum contraception included being older at delivery, having lower educational attainment, postpartum Medicaid or CHIP coverage, or being uninsured. Furthermore, we found notable differences by race and ethnicity, with higher odds of not using contraception among NH-Black, American Indian or Alaska Native, and Asian or Pacific Islander postpartum people relative to NH-white people.

Our findings were consistent with previous literature and provide an important update to a 2007 study that also used PRAMS free-text responses for contraceptive nonuse (Nettleman et al., 2007). Our analysis found the second most common free-text response for not using contraception was related to infertility, in line with their study (Nettleman et al., 2007). Their study found many respondents believed they could not become pregnant while breastfeeding, similar to the approximately 10% of free-text responses we identified that mentioned breastfeeding. Our results update this literature, offering insight into the family planning decisions of postpartum people after implementation of the ACA and the COVID-19 pandemic.

The 2022 US Supreme Court *Dobbs v Jackson Women’s Health Organization* decision ended the federal right to abortion with implications for contraceptive access. A growing body of literature has documented changes in contraceptive access in the wake of the Dobbs decision (Ellison et al., 2024; Flink-Bochacki et al., 2024; Liang et al., 2023; Muller et al., 2025; Qato et al., 2024). Using prescription data, one study found a decline in oral contraceptives, especially in states with the most restrictive abortion policies (Qato et al., 2024). Other studies using medical record data observed increases in long-acting reversible contraceptives (Muller et al., 2025) and increases in permanent contraception (e.g., tubal ligation, vasectomies) among adults between 18 and 30, especially for female patients (Ellison et al., 2024). A single site study in Michigan also initially observed an increase in tubal sterilization, but results did not persist 6-months after the Dobbs decision (Liang et al., 2023). Researchers at the Guttmacher Institute used survey data from four states and found decreases in sexual activity, increases in condom use, and increases in barriers to contraceptive access including poorer quality contraceptive care (Kavanaugh & Friedrich-Karnik, 2024). Collectively, this nascent literature suggests access to contraception has deteriorated since the Dobbs decision, which in turn impacts birth spacing and prevalence of rapid repeat pregnancies.

Both the Healthy People 2030 goal of improving pregnancy spacing and a recommendation by the American College of Obstetricians and Gynecologists (ACOG) to avoid interpregnancy intervals shorter than 6 months highlight the need for access to family planning services and contraceptive counseling after childbirth (Louis et al., 2019). Our findings suggest a need to strengthen access to routine postpartum visits, where contraceptive counseling is supposed to occur. Many respondents reported long wait times, scheduling difficulties, and lack of transportation as barriers to attending their follow-up appointments, particularly during the pandemic. Rethinking delivery approaches to postpartum care may improve rates of postpartum visits. For example, offering postpartum contraceptive services at the infant well-child visit could improve rates of postpartum contraception when birthing people are less likely to miss infant visits. The American Academy of Pediatrics (AAP) recommends screening for postpartum depression during the infant well-child visit instead of relying on the postpartum visit, which indicates how integral the well-child visit may be (ACOG, 2018).

Providers do not need to wait until the postpartum period to conduct contraceptive counseling. The ACOG recommends postpartum contraceptive counseling begin in the prenatal period and that postpartum visits are scheduled during prenatal care or before hospital discharge (ACOG, 2018). Similarly, IUD insertion, hysterectomies, and tubal sterilization are all highly effective methods that can happen before hospital discharge depending on insurance coverage. Despite ACA coverage increases, insurance barriers continue to affect postpartum people’s access to family planning services. In particular, current Medicaid policies create barriers to postpartum family planning through (1) not covering hysterectomies for the purpose of sterilization (Sterilization by Hysterectomy, 1982) and (2) its 30-day minimum waiting period for tubal sterilization procedures (Sterilization of a Mentally Competent Individual Aged 21 or Older, 1978). Our findings that respondents with Medicaid or CHIP were less likely to use postpartum contraception compared to privately insured people may be an indication of policy barriers. Researchers have called on reform to barriers in Medicaid policies to improve access to highly effective contraceptive methods (Borrero et al., 2014; Hahn et al., 2019; Morris et al., 2019).

Actions to increase postpartum access to all family planning options, including supporting birthing people who prefer not to use contraception or hope to quickly become pregnant again, meaningfully promotes reproductive autonomy and reproductive justice (ACOG, 2025; McAllister et al., 2023). Providing education and resources on safely becoming pregnant again, navigating sex without birth control, and ensuring the availability of all contraceptive methods can empower individuals to make informed decisions regarding if and when to become pregnant again, fostering autonomy and control over reproductive choices (Bullington et al., 2022; Sinai & Cachan, 2012). A 2025 statement by ACOG emphasizes the importance of high-quality contraceptive counseling naming reproductive autonomy, timeliness of access and referrals, accurate information, and promotion of the full scope of contraceptive methods as integral considering the post-Dobbs landscape (ACOG, 2025). Addressing multi-level barriers to postpartum contraception including at the provider, structural, and policy level can contribute to a more equitable and just reproductive healthcare system (Gibson et al., 2024; Tran et al., 2026). Our findings suggest that additional policy and clinical-level interventions are necessary to reduce current barriers and promote reproductive justice.

This study has several limitations. This analysis relies upon data collected during the COVID-19 pandemic. The pandemic may have differentially affected responses, and the data could be missing perspectives from people who were unable to participate. However, 2020–2021 PRAMS only includes states that have a minimum response threshold of 50% (Centers for Disease Control and Prevention, 2024). Importantly, the study years preclude the 2022 Dobbs decision, which we would expect to both increase demand and utilization of postpartum contraception (Larkin et al., 2025). Still, the primary reasons for not using postpartum contraception ranged from concerns about contraceptive side effects to not wanting to use contraception to appointment processes, which likely remain common reasons to not using contraception in the postpartum period. Additionally, PRAMS includes respondents typically 2–6 months after childbirth, and respondents may receive contraceptive counseling and care later in the postpartum period. However, birthing people should have attended at least one postpartum visit by 4 months (ACOG, 2018), so this analysis likely captures the period during which most postpartum contraception would be provided.

Our study highlights several factors—ranging from clinical education needs to barriers such as appointment availability, timing, and cost—that influence reasons for using contraception in the postpartum period, with disparities across demographic groups that could be contributing to reproductive health inequities. Our findings provide new insight into the family planning decisions of postpartum people considering the COVID-19 pandemic and ACA implementation. By exploring the reasons why postpartum people were not taking actions to prevent pregnancy, this research can inform provider practices and policies regarding access to reproductive healthcare, contraception, and family planning services. Given the hostile reproductive policy environment since the 2022 Dobbs decision, understanding contraceptive decision-making is critical to ensuring the health and well-being of all birthing people.

## Electronic Supplementary Material

Below is the link to the electronic supplementary material.

Supplementary Material 1

## Data Availability

PRAMS data is no longer available as of 2026, but was previously available through the CDC.

## Code Availability

All analyses were conducted in 2025 and 2026 using Stata v.18.5.
