# Supplementary material for: Barriers and Needs for Postpartum Contraception
Source: Matern Child Health J. 2026 May 4;30(5):681–91. doi: 10.1007/s10995-026-04265-0 (PMC13190350; doi:10.1007/s10995-026-04265-0)
Supplement: Supplementary file 1 — Supplementary Material 1 [file 10995_2026_4265_MOESM1_ESM.docx]

**Supplementary Material**

**Supplementary Material 1. States included in the sample and annual weighted response rate, 2019-2021**

| State | 2019 weighted response rate | 2020 weighted response rate | 2021 weighted response rate |
| --- | --- | --- | --- |
| Alabama | 56.4 | 55.2 | 49.7 |
| Arizona | 45.7 | 53.0 | 42.3 |
| Arkansas | 55.6 | 58.3 | 52.9 |
| Colorado | 59.4 | 63.3 | 60.2 |
| Connecticut | 55.7 | 63.2 | 58.9 |
| Delaware | 57.4 | 53.7 | 52.1 |
| District of Columbia | 51.9 | 58.6 | 54.6 |
| Florida | 51.1 | 55.6 | 42.7 |
| Georgia | 61.0 | 53.1 | 50.1 |
| Hawaii | 55.5 | 62.3 | 56.4 |
| Illinois | 59.3 | 61.1 | 56.4 |
| Iowa | 49.6 | 50.5 | 44.4 |
| Kansas | 63.3 | 65.8 | 63.4 |
| Kentucky | 60.4 | 60.4 | 45.5 |
| Louisiana | 57.1 | 57.3 | 56.8 |
| Maine | 61.4 | 55.2 | 50.0 |
| Maryland | 50.5 | 49.5 | 44.1 |
| Massachusetts | 61.2 | 59.6 | 57.0 |
| Michigan | 55.6 | 59.2 | 57.0 |
| Minnesota | 54.7 | 54.2 | 52.1 |
| Mississippi | 65.0 | 60.4 | 54.4 |
| Missouri | 56.9 | 57.2 | 50.1 |
| Montana | 51.3 | 53.1 | 55.1 |
| Nebraska | 63.8 | 66.0 | 59.4 |
| New Hampshire | 51.4 | 51.9 | 42.5 |
| New Jersey | 65.3 | 64.8 | 56.3 |
| New Mexico | 66.6 | 61.7 | 62.2 |
| New York | 52.8 | 49.5 | 56.8 |
| New York City | 60.8 | 61.3 | 59.2 |
| North Dakota | 59.1 | 60.5 | 54.1 |
| Oklahoma | 48.1 | 40.1 | 53.0 |
| Oregon | 69.3 | 64.2 | 56.8 |
| Pennsylvania | 58.2 | 60.3 | 56.1 |
| South Dakota | 68.1 | 66.8 | 65.9 |
| Tennessee | 54.4 | 55.5 | 54.8 |
| Utah | 72.8 | 66.9 | 58.2 |
| Vermont | 62.4 | 66.1 | 62.9 |
| Virginia | 55.5 | 57.6 | 49.8 |
| Washington | 64.1 | 64.4 | 60.6 |
| West Virginia | 49.2 | 50.4 | 51.0 |
| Wisconsin | 59.7 | 64.1 | 55.8 |
| Wyoming | 55.0 | 51.4 | 54.2 |

Notes: 40 states, New York City, and District of Columbia included in the sample. Alaska and Puerto Rico are excluded from the sample due to differing income categories. Weighted response rates are provided by PRAMS.

**Supplementary Material 2. Sensitivity analysis of the marginal effects of the characteristics associated with not taking family planning actions in the postpartum period, 2019-2021**

|  | 2019  (N = 27,251) | 2020-2021  (N = 88,664) |
| --- | --- | --- |
| Demographic characteristics | Predicted probability | Predicted probability |
| Age |  |  |
| 19 or younger | 17.51 | 17.16 |
|  | (13.58 - 21.44) | (15.21 - 19.12) |
| 20-24 | 19.84 | 22.48 |
|  | (17.76 - 21.92) | (21.31 - 23.65) |
| 25-29 | 19.82 | 22.75 |
|  | (18.4 - 21.24) | (21.91 - 23.6) |
| 30-34 | 19.88 | 23.12 |
|  | (18.47 - 21.29) | (22.27 - 23.96) |
| 35-39 | 25.82 | 26.78 |
|  | (23.67 - 27.97) | (25.56 - 28) |
| 40+ | 30.09 | 32.76 |
|  | (25.19 - 34.98) | (30.13 - 35.39) |
| Educational attainment |  |  |
| More than high school | 19.58 | 25.62 |
|  | (18.61 - 20.55) | (24.69 - 26.55) |
| High school education or less | 23.68 | 22.34 |
|  | (21.96 - 25.39) | (21.74 - 22.93) |
| Race/Ethnicity |  |  |
| White, non-Hispanic | 20.14 | 21.90 |
|  | (19.07 - 21.21) | (21.28 - 22.53) |
| Black, non-Hispanic | 24.45 | 27.53 |
|  | (22.22 - 26.69) | (26.28 - 28.78) |
| Hispanic or Latino | 16.90 | 21.83 |
|  | (15.02 - 18.77) | (20.69 - 22.97) |
| Asian or Pacific Islander | 30.37 | 32.40 |
|  | (26.85 - 33.88) | (30.45 - 34.35) |
| American Indian or Alaska Native | 20.53 | 27.75 |
|  | (16.24 - 24.82) | (24.02 - 31.48) |
| Other | 28.73 | 26.54 |
|  | (23.45 - 34.02) | (24.14 - 28.95) |
| Marital status |  |  |
| Married | 21.03 | 23.26 |
|  | (19.91 - 22.16) | (22.62 - 23.9) |
| Not Married | 20.86 | 23.90 |
|  | (19.32 - 22.4) | (23.04 - 24.76) |
| Number of prior births |  |  |
| 0 | 24.29 | 26.89 |
|  | (22.88 - 25.71) | (26.08 - 27.7) |
| 1 | 19.39 | 22.47 |
|  | (18.09 - 20.7) | (21.68 - 23.26) |
| 2 | 19.11 | 19.30 |
|  | (17.17 - 21.06) | (18.27 - 20.34) |
| 3 or more | 17.69 | 21.37 |
|  | (15.55 - 19.83) | (20.06 - 22.68) |
| Postpartum health insurance |  |  |
| Private | 19.67 | 22.35 |
|  | (18.46 - 20.88) | (21.61 - 23.1) |
| Medicaid or CHIP | 21.82 | 24.84 |
|  | (20.04 - 23.61) | (23.93 - 25.76) |
| Uninsured | 24.15 | 24.81 |
|  | (21.20 - 27.11) | (22.98 - 26.63) |
| Other | 22.39 | 23.79 |
|  | (18.51 - 26.27) | (21.60 - 25.98) |
| Income |  |  |
| 138% FPL and below | 20.41 | 22.62 |
|  | (18.72 - 22.09) | (21.68 - 23.57) |
| 139% - 200% FPL | 19.73 | 22.81 |
|  | (17.36 - 22.11) | (21.34 - 24.28) |
| Above 200% FPL | 19.78 | 23.01 |
|  | (18.19 - 21.37) | (22.03 - 23.99) |

Notes: Authors’ sensitivity analysis of 2019-2021 Pregnancy Risk Assessment Monitoring System data from 40 states, New York City, and the District of Columbia. Marginal effects presented with robust 95% confidence intervals.

**Supplementary Material 3. Sensitivity analysis of the marginal effects of the characteristics associated with not taking family planning actions in the postpartum period using health insurance at delivery, 2019-2021**

|  | 2019  (N = 27,251) | 2020-2021  (N = 88,664) |
| --- | --- | --- |
| Demographic characteristics | Predicted probability | Predicted probability |
| Age |  |  |
| 19 or younger | 17.63 | 17.17 |
|  | (13.65 - 21.61) | (15.21 - 19.13) |
| 20-24 | 19.90 | 22.54 |
|  | (17.80 - 22.00) | (21.37 - 23.71) |
| 25-29 | 19.83 | 22.76 |
|  | (18.41 - 21.25) | (21.92 - 23.61) |
| 30-34 | 19.87 | 23.09 |
|  | (18.46 - 21.28) | (22.25 - 23.94) |
| 35-39 | 25.73 | 26.75 |
|  | (23.58 - 27.88) | (25.53 - 27.97) |
| 40+ | 29.92 | 32.66 |
|  | (25.01 - 34.84) | (30.04 - 35.29) |
| Educational attainment |  |  |
| More than high school | 23.87 | 25.56 |
|  | (22.12 - 25.61) | (24.63 - 26.48) |
| High school education or less | 19.49 | 22.37 |
|  | (18.52 - 20.46) | (21.77 - 22.96) |
| Race/Ethnicity |  |  |
| White, non-Hispanic | 20.06 | 21.93 |
|  | (18.99 - 21.12) | (21.31 - 22.55) |
| Black, non-Hispanic | 24.40 | 27.57 |
|  | (22.15 - 26.64) | (26.32 - 28.81) |
| Hispanic or Latino | 17.19 | 21.72 |
|  | (15.31 - 19.07) | (20.60 - 22.84) |
| Asian or Pacific Islander | 30.37 | 32.44 |
|  | (26.85 - 33.9) | (30.49 - 34.4) |
| American Indian or Alaska Native | 20.68 | 27.73 |
|  | (16.35 - 25.01) | (24.07 - 31.4) |
| Other | 28.74 | 26.55 |
|  | (23.33 - 34.15) | (24.14 - 28.96) |
| Marital status |  |  |
| Married | 20.93 | 23.18 |
|  | (19.81 - 22.05) | (22.54 - 23.81) |
| Not Married | 21.02 | 24.02 |
|  | (19.48 - 22.57) | (23.17 - 24.88) |
| Number of prior births |  |  |
| 0 | 24.24 | 26.90 |
|  | (22.83 - 25.66) | (26.09 - 27.71) |
| 1 | 19.41 | 22.48 |
|  | (18.10 - 20.71) | (21.69 - 23.27) |
| 2 | 19.12 | 19.30 |
|  | (17.17 - 21.07) | (18.27 - 20.33) |
| 3 or more | 17.77 | 21.31 |
|  | (15.63 - 19.9) | (20.01 - 22.61) |
| Health insurance at delivery |  |  |
| Private | 20.29 | 22.46 |
|  | (19.05 - 21.53) | (21.76 - 23.16) |
| Medicaid | 21.67 | 24.58 |
|  | (20.05 - 23.3) | (23.71 - 25.46) |
| Uninsured | 24.23 | 27.10 |
|  | (19.18 - 29.27) | (24.31 - 29.9) |
| Other | 22.70 | 27.10 |
|  | (15.88 - 29.53) | (22.93 - 31.28) |
| Income |  |  |
| 138% FPL and below | 20.64 | 22.73 |
|  | (18.93 - 22.34) | (21.78 - 23.67) |
| 139% - 200% FPL | 19.94 | 22.91 |
|  | (17.55 - 22.34) | (21.44 - 24.38) |
| Above 200% FPL | 19.63 | 22.99 |
|  | (18.05 - 21.22) | (22.00 - 23.97) |

Notes: Authors’ sensitivity analysis of 2019-2021 Pregnancy Risk Assessment Monitoring System data from 40 states, New York City, and the District of Columbia. Health insurance at delivery used in analysis instead of postpartum health insurance. Marginal effects presented with robust 95% confidence intervals.

**Supplementary Material 4. Proportions of the Study Sample Not Using** **Postpartum Contraception by Demographic Characteristics, 2019-2021**

Examining rates of not using postpartum contraception across sample characteristics, we found higher rates among birthing people who had educational attainment of a high school education or less, were age 40 or older at delivery, were non-Hispanic Asian or Pacific Islander, were unmarried, had no previous births, and had postpartum health insurance that was not private coverage.

|  | Weighted Proportion |
| --- | --- |
| Age (in years) |  |
| 19 or younger | 22.21 (20.26 - 24.28) |
| 20-24 | 24.03 (23.04 - 25.06) |
| 25-29 | 21.91 (21.20 - 22.65) |
| 30-34 | 21.33 (20.66 - 22.01) |
| 35-39 | 25.00 (24.04 - 25.98) |
| 40+ | 30.77 (28.58 - 33.05) |
| Educational attainment |  |
| High school education or less | 24.52 (23.82 - 25.22) |
| More than high school education | 22.00 (21.54 - 22.48) |
| Race/Ethnicity |  |
| White, non-Hispanic | 21.11 (20.61 - 21.63) |
| Black, non-Hispanic | 27.09 (26.05 - 28.15) |
| Hispanic or Latino | 21.46 (20.51 - 22.43) |
| Asian or Pacific Islander | 32.57 (30.90 - 34.28) |
| American Indian or Alaska Native | 26.60 (23.63 - 29.79) |
| Other | 26.91 (24.73 - 29.20) |
| Marital status |  |
| Married | 22.18 (21.70 - 22.66) |
| Not Married | 24.09 (23.43 - 24.76) |
| Number of prior births |  |
| 0 | 25.36 (24.71 - 26.01) |
| 1 | 21.58 (20.91 - 22.26) |
| 2 | 19.85 (18.95 - 20.79) |
| 3 or more | 22.70 (21.59 - 23.85) |
| Postpartum health insurance |  |
| Private | 21.89 (21.36 - 22.42) |
| Medicaid or CHIP | 24.05 (23.38 - 24.74) |
| Uninsured | 23.99 (22.56 - 25.47) |
| Other | 23.46 (21.62 - 25.41) |
| Income |  |
| 138% FPL and below | 23.10 (22.37 - 23.84) |
| 139% - 200% FPL | 22.53 (21.30 - 23.81) |
| Above 200% FPL | 21.67 (20.88 - 22.48) |

Notes: Authors’ analysis of 2019-2021 Pregnancy Risk Assessment Monitoring System data from 40 states, New York City, and the District of Columbia. Weighted proportions are presented. 95% confidence intervals are in parentheses.

**Supplementary Material 5. Characteristics Associated with Not Taking Family Planning Actions in the Postpartum Period Adjusting for State, 2019-2021**

In sensitivity analyses including state fixed effects and state clustered standard errors to account for state-level variation, we found results that showed similar patterns of association between demographics and not taking family planning actions in the postpartum period as in main models. In both 2019 and 2020-21, associations by education, race and ethnicity, marital status, age, number of prior births, and income were consistent with main model findings. In contrast to main models, we did not find any evidence that uninsured postpartum people had higher odds of not taking family planning actions in 2020-21 compared to privately insured postpartum people after adjusting for state.

|  | 2019  (N = 27,251) | 2020-2021  (N = 88,664) |
| --- | --- | --- |
| Demographic characteristics | Odds Ratio | Odds Ratio |
| Age |  |  |
| 19 or younger | Reference | Reference |
| 20-24 | 1.11 | 1.38*** |
|  | (0.84 - 1.47) | (1.18 - 1.60) |
| 25-29 | 1.08 | 1.38*** |
|  | (0.84 - 1.40) | (1.17 - 1.62) |
| 30-34 | 1.07 | 1.38** |
|  | (0.79 - 1.46) | (1.14 - 1.67) |
| 35-39 | 1.49* | 1.67*** |
|  | (1.09 - 2.04) | (1.42 - 1.96) |
| 40+ | 1.90*** | 2.22*** |
|  | (1.45 - 2.48) | (1.83 - 2.70) |
| Educational attainment |  |  |
| More than high school | Reference | Reference |
| High school education or less | 1.29*** | 1.19*** |
|  | (1.17 - 1.42) | (1.09 - 1.29) |
| Race/Ethnicity |  |  |
| White, non-Hispanic | Reference | Reference |
| Black, non-Hispanic | 1.28*** | 1.34*** |
|  | (1.12 - 1.46) | (1.19 - 1.50) |
| Hispanic or Latino | 0.79* | 0.96 |
|  | (0.65 - 0.97) | (0.87 - 1.05) |
| Asian or Pacific Islander | 1.56** | 1.62*** |
|  | (1.20 - 2.04) | (1.40 - 1.88) |
| American Indian or Alaska Native | 1.06 | 1.45** |
|  | (0.77 - 1.47) | (1.16 - 1.81) |
| Other | 1.59*** | 1.28** |
|  | (1.28 - 1.97) | (1.09 - 1.50) |
| Marital status |  |  |
| Married | Reference | Reference |
| Not Married | 0.98 | 1.02 |
|  | (0.85 - 1.14) | (0.94 - 1.11) |
| Number of prior births |  |  |
| 0 | Reference | Reference |
| 1 | 0.76*** | 0.79*** |
|  | (0.67 - 0.85) | (0.74 - 0.84) |
| 2 | 0.75** | 0.65*** |
|  | (0.62 - 0.90) | (0.61 - 0.69) |
| 3 or more | 0.68** | 0.75*** |
|  | (0.55 - 0.86) | (0.68 - 0.83) |
| Postpartum health insurance |  |  |
| Private | Reference | Reference |
| Medicaid or CHIP | 1.10 | 1.14** |
|  | (0.94 - 1.29) | (1.05 - 1.24) |
| Uninsured | 1.33* | 1.18 |
|  | (1.04 - 1.69) | (0.98 - 1.42) |
| Other | 1.11 | 1.04 |
|  | (0.88 - 1.38) | (0.94 - 1.15) |
| Income |  |  |
| 138% FPL and below | Reference | Reference |
| 139% - 200% FPL | 0.97 | 1.01 |
|  | (0.81 - 1.15) | (0.93 - 1.11) |
| Above 200% FPL | 0.96 | 1.02 |
|  | (0.83 - 1.12) | (0.94 - 1.11) |

Notes: Authors’ analysis of 2019-2021 Pregnancy Risk Assessment Monitoring System data from 40 states, New York City, and the District of Columbia. Weighted odds ratios presented with robust 95% confidence intervals. *p<0.05, **p<0.01, ***p<0.001

**Supplementary Material 6. Characteristics Associated with Not Taking Family Planning Actions in the Postpartum People by Postpartum Insurance, 2019-2021**

Supplementary Material 6 presents multivariable logistic regression results for stratified models by postpartum insurance. Overall, especially among Medicaid and privately insured postpartum people, we observe results that showed similar patterns of association between demographic characteristics and not taking family planning actions as in main models. For those with Medicaid or private insurance, in both 2019 and 2020-21, having a prior birth was associated with lower odds of not using postpartum contraception compared to primiparous respondents. For those with private insurance, in both 2019 and 2020-21, having a high school education or less was associated with higher odds of not using postpartum contraception compared to those with more than a high school education. For uninsured people, only those who identify as Hispanic or Latine were consistently associated (2019 and 2020-21) with lower odds of not using postpartum contraception compared to NH-white respondents.

|  | Medicaid | | Private | | Uninsured | |
| --- | --- | --- | --- | --- | --- | --- |
|  | 2019  (N = 8,549) | 2020-2021  (N = 33,361) | 2019  (N=14,248) | 2020-2021  (N=42,849) | 2019  (N=2,816) | 2020-2021  (N=7,667) |
| Demographic characteristics | Odds Ratio | Odds Ratio | Odds Ratio | Odds Ratio | Odds Ratio | Odds Ratio |
| Age |  |  |  |  |  |  |
| 19 or younger | Reference | Reference | Reference | Reference | Reference | Reference |
| 20-24 | 1.23 | 1.39*** | 0.85 | 1.34 | 1.17 | 1.26 |
|  | (0.86 - 1.78) | (1.16 - 1.66) | (0.42 - 1.69) | (0.88 - 2.03) | (0.52 - 2.61) | (0.80 - 1.97) |
| 25-29 | 1.38 | 1.45*** | 0.81 | 1.46 | 1.01 | 1.21 |
|  | (0.94 - 2.01) | (1.20 - 1.76) | (0.41 - 1.61) | (0.96 - 2.20) | (0.44 - 2.30) | (0.76 - 1.92) |
| 30-34 | 1.48 | 1.45*** | 0.82 | 1.58* | 1.04 | 1.13 |
|  | (0.99 - 2.21) | (1.18 - 1.78) | (0.41 - 1.64) | (1.04 - 2.39) | (0.44 - 2.44) | (0.70 - 1.84) |
| 35-39 | 1.72* | 1.61*** | 1.28 | 2.03*** | 1.30 | 1.22 |
|  | (1.10 - 2.70) | (1.29 - 2.01) | (0.64 - 2.57) | (1.34 - 3.08) | (0.52 - 3.20) | (0.73 - 2.05) |
| 40+ | 2.05* | 1.67** | 1.55 | 3.17*** | 3.58* | 1.57 |
|  | (1.09 - 3.86) | (1.22 - 2.29) | (0.74 - 3.24) | (2.04 - 4.90) | (1.26 - 10.19) | (0.84 - 2.93) |
| Educational attainment |  |  |  |  |  |  |
| More than high school | Reference | Reference | Reference | Reference | Reference | Reference |
| High school education or less | 1.18 | 1.19*** | 1.25* | 1.23*** | 1.43 | 1.26* |
|  | (0.98 - 1.43) | (1.08 - 1.30) | (1.01 - 1.55) | (1.09 - 1.38) | (1.00 - 2.04) | (1.00 - 1.58) |
| Race/Ethnicity |  |  |  |  |  |  |
| White, non-Hispanic | Reference | Reference | Reference | Reference | Reference | Reference |
| Black, non-Hispanic | 1.25* | 1.28*** | 1.36* | 1.52*** | 0.91 | 0.95 |
|  | (1.01 - 1.55) | (1.16 - 1.42) | (1.08 - 1.71) | (1.34 - 1.74) | (0.56 - 1.47) | (0.69 - 1.30) |
| Hispanic or Latino | 0.82 | 1.01 | 0.89 | 1.16* | 0.50*** | 0.62*** |
|  | (0.63 - 1.06) | (0.90 - 1.14) | (0.70 - 1.14) | (1.02 - 1.32) | (0.33 - 0.74) | (0.48 - 0.79) |
| Asian or Pacific Islander | 1.21 | 1.73*** | 2.16*** | 1.76*** | 0.37 | 1.28 |
|  | (0.79 - 1.86) | (1.41 - 2.11) | (1.75 - 2.65) | (1.57 - 1.98) | (0.13 - 1.04) | (0.78 - 2.10) |
| American Indian or Alaska Native | 0.97 | 1.34* | 1.07 | 1.86 | 0.82 | 0.93 |
|  | (0.66 - 1.44) | (1.03 - 1.73) | (0.43 - 2.65) | (0.87 - 3.98) | (0.51 - 1.31) | (0.71 - 1.22) |
| Other | 1.20 | 1.08 | 1.59* | 1.48*** | 1.66 | 1.22 |
|  | (0.77 - 1.87) | (0.89 - 1.31) | (1.11 - 2.26) | (1.23 - 1.80) | (0.76 - 3.64) | (0.73 - 2.03) |
| Marital status |  |  |  |  |  |  |
| Married | Reference | Reference | Reference | Reference | Reference | Reference |
| Not Married | 0.90 | 0.94 | 1.08 | 1.11 | 0.92 | 1.04 |
|  | (0.73 - 1.10) | (0.85 - 1.03) | (0.88 - 1.34) | (0.99 - 1.25) | (0.65 - 1.30) | (0.85 - 1.27) |
| Number of prior births |  |  |  |  |  |  |
| 0 | Reference | Reference | Reference | Reference | Reference | Reference |
| 1 | 0.73** | 0.81*** | 0.73*** | 0.75*** | 0.92 | 0.88 |
|  | (0.57 - 0.92) | (0.73 - 0.91) | (0.63 - 0.84) | (0.69 - 0.81) | (0.61 - 1.39) | (0.68 - 1.14) |
| 2 | 0.72* | 0.71*** | 0.61*** | 0.60*** | 0.86 | 0.67* |
|  | (0.55 - 0.94) | (0.62 - 0.81) | (0.49 - 0.74) | (0.53 - 0.67) | (0.49 - 1.52) | (0.49 - 0.91) |
| 3 or more | 0.60*** | 0.74*** | 0.65** | 0.70*** | 0.70 | 0.83 |
|  | (0.45 - 0.80) | (0.64 - 0.86) | (0.49 - 0.87) | (0.59 - 0.82) | (0.41 - 1.21) | (0.62 - 1.13) |
| Income |  |  |  |  |  |  |
| 138% FPL and below | Reference | Reference | Reference | Reference | Reference | Reference |
| 139% - 200% FPL | 0.83 | 0.99 | 1.30 | 1.04 | 0.72 | 0.90 |
|  | (0.64 - 1.09) | (0.87 - 1.13) | (0.92 - 1.85) | (0.85 - 1.29) | (0.44 - 1.15) | (0.66 - 1.22) |
| Above 200% FPL | 0.86 | 0.97 | 1.10 | 1.05 | 0.77 | 0.90 |
|  | (0.65 - 1.14) | (0.85 - 1.10) | (0.82 - 1.48) | (0.89 - 1.24) | (0.47 - 1.26) | (0.67 - 1.20) |

Notes: Authors’ analysis of 2019-2021 Pregnancy Risk Assessment Monitoring System data from 40 states, New York City, and the District of Columbia. Weighted odds ratios presented with robust 95% confidence intervals. *p<0.05, **p<0.01, ***p<0.001
